# A silver lining in the VAD sky? Impact of a silver dressing on left ventricular assist device-associated driveline infections

**DOI:** 10.1016/j.jhlto.2026.100556

**Published:** 2026-04-10

**Authors:** Sylvie Baudart, Liviu Klein

**Affiliations:** Advanced Heart Failure Comprehensive Care Center, University of California at San Francisco, San Francisco, CA

**Keywords:** Driveline, Infection, Ventricular assist device, Silver, Dressing, Chlorhexidine

## Abstract

**Background:**

Left ventricular assist devices (LVAD) constitute a mainstay of treatment for patients suffering from advanced heart failure. LVAD have been shown to significantly increase patient survival and quality of life. The therapy continues to be hampered by LVAD-specific complications that limit its wider appeal. Driveline infections (DLI) remain the Achilles’ heel of LVAD therapy, with persistent rates of DLI plaguing many patients. Several studies have been carried out to evaluate the effectiveness of driveline dressing protocols, but there is no consensus on specific driveline management, and no prospective trial has ever been carried out.

**Methods:**

We conducted a single-center prospective randomized trial of a driveline dressing protocol to evaluate the safety and efficacy of a weekly silver patch-based driveline dressing kit. The Control group protocol included every 3 days changes with chlorhexidine cleansing, an occlusive dressing, and an anchoring device. The Silver group protocol included weekly dressing changes with chlorhexidine cleansing, a non-activated silver patch, an occlusive dressing, and an anchoring device. Twenty-three patients were enrolled in the study, with 11 in the control group and 12 in the silver group. The endpoints of the study were the occurrence of the first DLI, time to first DLI, and total number of DLI at 18 months. We used SAS to assess the significance of the primary endpoint.

**Results:**

At an average 18-month follow-up, the first DLI rate (0.061 vs 0.377 events per patient year [EPPY], *p* = 0.01) as well as the total DLI rate (0.061 vs 0.754 EPPY, *p* = 0.002) was lower in the intervention arm. The mean time to first DLI was longer in the intervention arm (432 vs 187 days, *p* = 0.05).

**Conclusion:**

A weekly driveline dressing protocol that includes a non-activated silver patch may provide protection against driveline infections.

## Graphical Abstract

**Figure:**
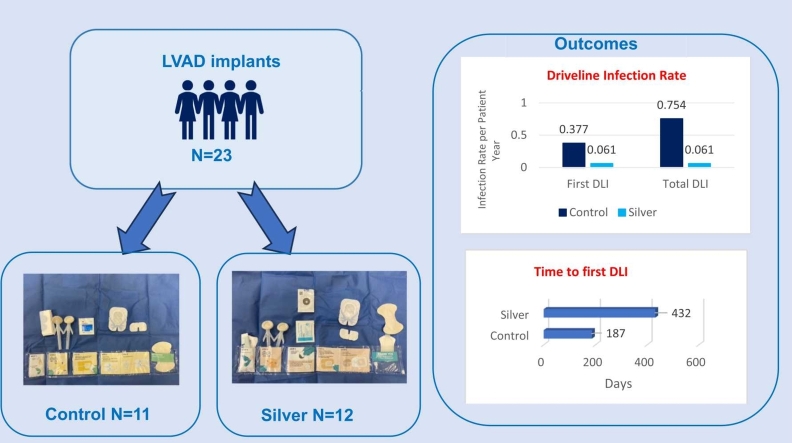

## Background

The advent of durable left ventricular assist devices (LVAD) has significantly shifted the treatment paradigm for end-stage heart failure by increasing patients’ survival and most importantly, allowing them a vastly improved quality of life at home.^1^, ^2^ Despite many advances in surgical technique and medical management, LVAD therapy remains hampered by LVAD-specific complications that limit its wider appeal.^3^ Advances in pump technology coupled with refined medical management protocols have reduced hemocompatibility-related adverse events such as pump thrombosis, stroke, and gastrointestinal bleeding. However, infections and more specifically, driveline infections (DLI) continue to complicate and shorten patient’s life on LVAD therapy. Infected LVAD patients are at higher risk of stroke, death, and have worst post-transplant outcomes.^3^, ^4^ These patients also face a worse quality of life with repeated admissions, prolonged antibiotic therapy, surgical debridement, and worsened functional status. The DLI rates vary vastly amongst LVAD centers (10%-50%), and DLI prevention protocols are not consistent.^4^, ^5^, ^6^ Several studies have been carried out to evaluate the effectiveness of driveline dressing protocols, but there is no consensus or widely accepted standardized approach to driveline management beyond using a standardized protocol, using an anchoring device to immobilize the driveline and an occlusive dressing to over the insertion site.^7^, ^8^, ^9^, ^10^ To date there has only been one prospective study^11^ and no prospective randomized controlled studies evaluating the effectiveness of a particular driveline dressing protocol. While many different dressing materials have been used, there is mounting evidence that silver-based dressing might be efficacious for both routine infection prevention and post-traumatic prophylaxis.^11^, ^12^, ^13^

We posited that silver-based dressing with weekly changes will reduce the rate of DLI while improving patients’ comfort and quality of life. Moreover, we hope that a prospective randomized controlled trial, the gold standard for treatment evaluation, will catalyze the standardization of dressing change protocols across LVAD centers. To this end, we conducted a prospective randomized study of a weekly silver patch-based dressing kit to evaluate its safety and efficacy on preventing DLI.

## Methods

The study was designed as a prospective randomized controlled trial of a driveline management protocol (NCT05163392). This was an IRB-approved single-center study, and the work was supported by a grant from the International Consortium of Circulatory Assist Clinicians.

### Study design

We included all adult patients (age greater than 18 years) undergoing LVAD implantation at our institution between January 2022 and December 2023 and excluded patients with a history of sternal wound infection and implantation secondary to exchange necessitated by LVAD components infection.

Participants were then randomly assigned to the 2 arms of the study with different driveline dressing kits. In the controlled arm of the study, patients were assigned to our institution standard kit. Patients in the intervention arm of the study were assigned to a newly designed dressing kit that contained a silver patch.

Manufacturer recommendations suggest utilization of a saline wipe to activate the silver patch. However, LVAD coordinators reported that the first 2 patients using a saline wipe to activate the silver showed signs of maceration at the driveline site, so the saline wipe was discontinued as we surmised that skin perspiration might be sufficient for activation, and bleeding or any drainage would further serve to activate the silver. With this approach, no further maceration at the driveline site was noted. Both arms of the study also contained a sensitive version of the kit in case participants developed an allergy to the cleansing agent, at which point they could be switched to the sensitive version of their assigned dressing kit.

**Figure 1:**
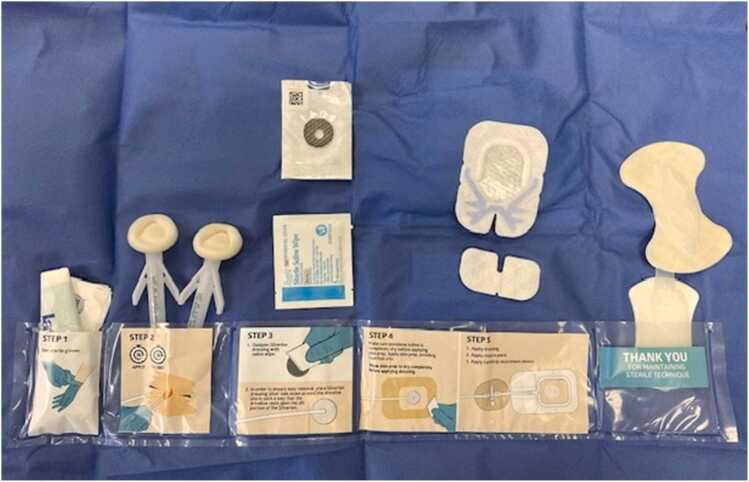
Silver kit used in the intervention arm of the study.
b. Sensitive skin dressing: a silver antimicrobial patch, povidone/iodine swabs for cleaning, an occlusive dressing with window, and sensitive driveline anchors. The driveline dressing change frequency is weekly.

The intervention arm kits contained:

a. Standard dressing: a silver antimicrobial patch, a saline wipe for activation of the silver patch (not used except for the first 2 patients), chlorhexidine (CHG) swabs for cleaning, occlusive dressing with window, and silicone driveline anchors. The driveline dressing change frequency is weekly (Figure 1).

**Figure 2:**
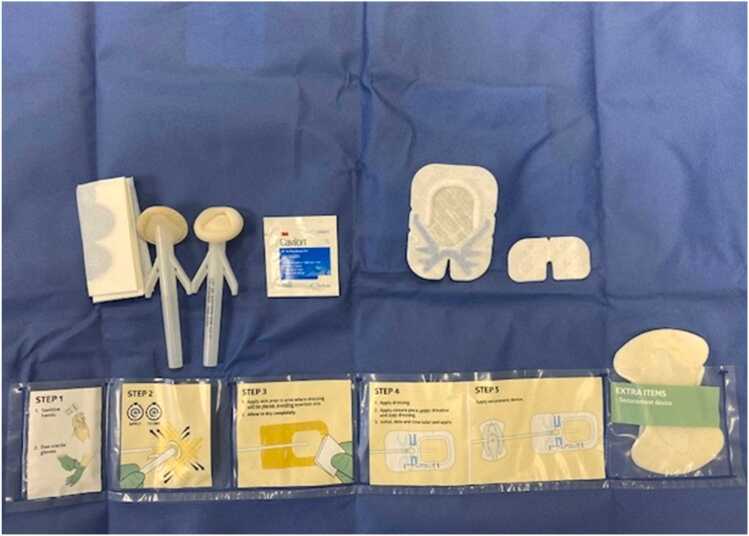
Standard kit used in the control arm of the study.
b. Sensitive skin dressing: No antimicrobial patch, povidone/iodine swabs for cleaning, an occlusive dressing with window, and sensitive driveline anchors. The driveline dressing change frequency is every 3 days.

The control arm kits contained:

a. Standard dressing: No antimicrobial patch, CHG swabs for cleaning, an occlusive dressing with window, and silicon driveline anchors. The driveline dressing change frequency is every 3 days (Figure 2).

Both kits were designed by the research team and assembled by Medline. The kits were available for purchase by UCSF and the DME companies that provide supplies to the patients. Patients were supplied dressing kits by their DME companies or by UCSF if their insurance did not cover driveline dressing supplies.

Both the VAD coordinators and VAD competent staff nurses were educated in the use of dressings for both arms of the study. Inpatient dressing changes were performed either by the VAD coordinators or the staff nurse assigned to the patient. Standard protocols dressings were available on the unit. Intervention protocol dressings were brought to the bedside by the VAD coordinators.

**Figure 3:**
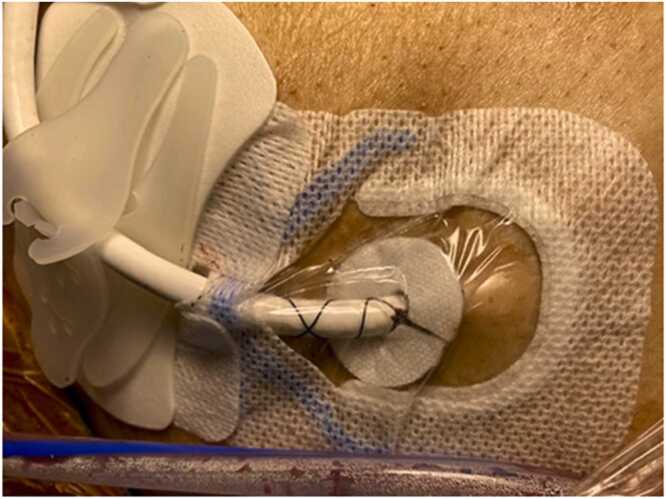
Silver dressing placed in the operating room after implant.

The dressings were applied immediately on Post op day 0 (Figure 3). The initial dressing was applied by the VAD coordinator present during the implant. Subsequent dressing changes were performed by the nurse assigned to the patient either in the ICU or the acute care unit. Staff could easily identify which dressing the patient was assigned to by observing the prior dressing, as the window of the occlusive dressing facilitates visualization of whether a silver patch is present.

Patients and caregivers were instructed in the use of the kits by the VAD coordinators during the VAD post implant teachings. Teaching was identical across the 2 arms of the study and consisted of a short didactic instruction and a demonstration of the driveline dressing change by the VAD coordinator. Skill acquisition and validation was accomplished through a guided driveline dressing change practice on a mannequin and at least a second demonstration on a mannequin. Additional practice demonstrations by the caregiver were performed as deemed necessary by the VAD coordinator. Final sign-off and skill validation were completed by assessment of the caregiver performing driveline dressing change on the patient.

The study aims were: 1) To evaluate the effectiveness of a silver-based driveline dressing protocol to reduce DLI as measured by DLI rate and time to first DLI; 2) To assess feasibility of a silver-based driveline dressing kit based on cost, comfort, and patient compliance.

DLI definition was applied using the STS INTERMACS definition of device-related infection. Drainage at the driveline site was assessed by a swabbing and sending for culture of the draining fluid. Because our center’s practice is to aggressively treat potential infection and to start antibiotics if signs and symptoms of infection are present even if a swab cannot be obtained prior to starting antibiotics, we defined infection as having significant drainage, whether or not the swab returned positive culture. Initial analysis was at a follow-up of 18 months post implant.

Surveys were sent to patients at 1 month, 6 months, and 12 months to assess patient-reported ease of use, comfort, compliance with the protocol, skin irritation, and dressing stability (Table 1).

**Table 1:** LVAD Silver Study Patient Survey

Question	Available responses
My driveline dressing is easy to use	Agree, somewhat agree, neither agree nor disagree, somewhat disagree, disagree
My driveline dressing is comfortable	Agree, somewhat agree, neither agree nor disagree, somewhat disagree, disagree
I follow my driveline dressing protocol timeline	Always, usually, sometimes, rarely, never
I experience irritation and/or itchiness at my driveline site	Always, usually, sometimes, rarely, never
My driveline dressing stays in place	Always, usually, sometimes, rarely, never

LVAD, left ventricular assist device.

### Data management and statistical analysis

Data was collected by the principal investigator and analyzed by our data analyst. Cost data was collected based on the number of kits sent to each patient. Patients were de-identified for all data collection and analysis. Data is presented as means with 95% confidence intervals for continuous variables, compared using t-tests. Regression analysis was conducted to test the treatment effect, controlling for body mass index, gender, and INTERMACS profile. All data analysis was performed using SAS version 9.4.

## Results

**Figure 4:**
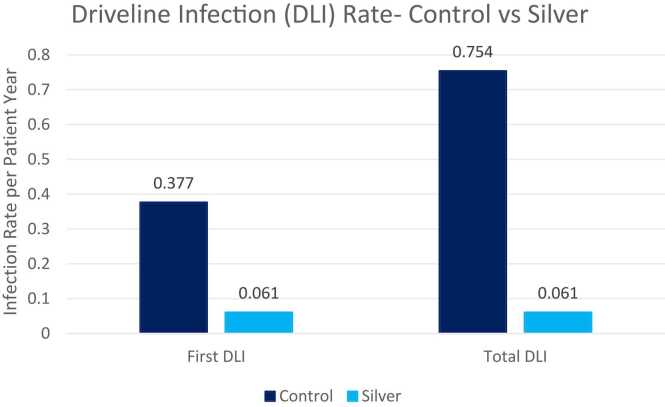
Driveline infection rate..

**Figure 5:**
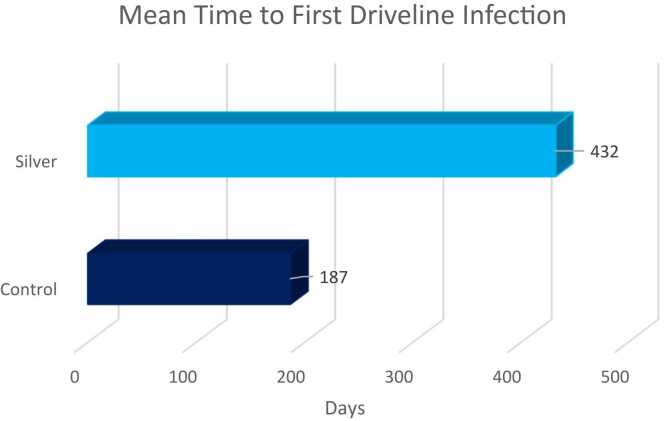
Mean time to driveline infection.

We enrolled 25 patients, 14 randomized to the intervention arm and 12 to the control arm. One patient in the control arm of the study who died during index hospitalization shortly after implant was excluded from analysis. Two patients in the intervention arms were excluded from the secondary analysis after the intervention protocol was updated to remove saline activation of the silver patch. The final analysis was conducted on 11 patients in the control group and 12 patients in the intervention group. Baseline characteristics did not differ significantly between the control and intervention group (mean age 50 vs 53 years, BMI 30 vs 36 Kg/m^2^, INTERMACS profile 1-2 54% vs 45%, Table 2). Only 3 female patients were enrolled in the study, and they were all randomly enrolled in the control group. The first DLI rate (0.061 vs 0.377 events per patient year [EPPY], *p* = 0.01), as well as the total DLI rate (0.061 vs 0.754 EPPY, *p* = 0.002) was lower in the intervention arm (Figure 4). There were no polymicrobial infections in the intervention arm. The mean time to first DLI was longer in the intervention arm (432 vs 187 days, *p* = 0.05) (Figure 5). There were no dermatitis reported in any arm of the study. We could not detect treatment effect in logistic or multivariate analysis secondary to the study low enrollment. Based on observed infection rate, an enrollment of 15 patients in each arm would have been necessary to detect a statistically significant difference in logistic or multivariate regression.

**Table 2:** Baseline Characteristics

Arm	Mean age at implant (years)	BMI kg/m^2^ (mean ± SD)	Male (%)	INTERMACS profile 1-2 (%)	Ethnicity (%) white/ hispanic/ black
Control	53	36.4 ± 7.1	75	45	27/ 18/ 45
Intervention	50	31.4 ± 7.9	100	54	25/ 58/ 17
*p* value	0.33	0.122	0.052	0.82	0.169

BMI, body mass index.

The monthly cost of the kit, accounting for kit usage, was significantly different between the two groups, with the silver kits showing a cost reduction by usage ($188.2 vs $327.1, *p* < 0.0001). Finally, there were no significant differences in comfort, ease of use, protocol adherence, or stability throughout the study between the two arms of the study, with high reports of satisfaction, ease of use, stability, and comfort in both groups at 1 month, 6 months, and 12 months. The control arm of the study group reported slightly higher rates of irritation and itchiness (Table 3).

**Table 3:** Summary of Survey Responses at 12-Month by Group

Question	Intervention group	Control group
My driveline dressing is easy to use	Agree/somewhat agree: 100%	Agree/somewhat agree: 100%
My driveline dressing is comfortable	Agree/somewhat agree: 100%	Agree/somewhat agree: 100%
I follow my driveline dressing protocol timeline	Always/usually: 100%	Always/usually: 100%
I experience irritation and/or itchiness at my driveline site	Rarely/never: 100%	Rarely/never: 64.6% Sometimes: 36.4%
My driveline dressing stays in place	Always/usually: 100%	Always/usually: 100%

## Discussion

In this prospective randomized controlled trial of a silver patch dressing kit for LVAD driveline dressing, we have found favorable findings indicating that adding a silver patch to a dressing kit and reducing the frequency of driveline dressing changes from every 3 days to every week may reduce the rate of first DLI and the overall rate of DLI. Our findings also indicate that the usage of the intervention kit may increase the time to first DLI. Unfortunately, we were not able to enroll enough patients to sufficiently power the study and detect significant effect in logistic or multivariate analysis. Both kits were very well tolerated, as evidenced by the overwhelming positive responses to questions of comfort, ease of use, stability, and compliance. Interestingly, a slightly higher rate of itchiness/irritation at the driveline site was reported in the control group. Patients who developed infections were more likely to report irritation/itchiness at the site, and we could not determine from the data any potential temporal relation between the increase in reported irritation and infection. We also could not determine whether the increase in reported irritation was secondary to infection or whether infection was more likely to develop in patients reporting irritation and itchiness who may in turn be more likely to scratch at or around their driveline site. Since both kits used chlorhexidine as cleaning agent and used the same occlusive dressing, it is unlikely that itchiness due to the dressing or cleaning agent is the main causal agent for developing irritation. Similarly, it is unlikely that the silver patch placed at the insertion site was somehow protecting patients from irritation. The frequency of dressing change could be a potential differentiator: Changing the dressing every 3 days could potentially cause increased peeling of the stratum corneum of the epidermis, causing increased irritation or microabrasion that could in turn lead to more infections.

As outlined earlier in the manuscript, the recommended usage of the silver patch is to moisten it with a saline wipe to activate the silver patch into releasing the silver cation. Due to maceration at the LVAD driveline insertion site in the first 2 patients following this approach, allowing for natural skin perspiration to activate the silver patch was undertaken instead. Furthermore, any drainage associated with infection would further serve to activate the silver in addition to baseline perspiration, so the saline wipe was not thought to be required for efficacy in our population. With our approach, no further skin maceration was noted. As the dressing protocol was modified, we excluded the two patients who had started the study with silver patch activation. Interestingly, both these patients later developed DLI (Table 4). It is possible that starting the weekly dressing with a moistened silver patch delays wound healing, eventually potentially increasing chances of infections. Creating a protocol in which weekly dressing would be initiated after initial site healing might mitigate the issue.

**Table 4:** Data on 2 Excluded Patients

Patient	Time to DLI (days)	Bacteria
A	256	Pseudomonas
B	103	MRSA

DLI, driveline infection.

Unfortunately, our sample gender distribution was heavily biased towards male patients (87%), and all three female patients were randomized to the control group. Infection rate amongst our female patient was 100%, and the small sample size limits further speculation about the significance of this finding. Future prospective studies investigating infection rates amongst different genders as well as caregiver gender and relationship to the patient may offer further insight. The cost analysis demonstrated that the silver kit is cost-effective despite the higher cost per kit ($30.9 for the control kit vs $36.7 for the silver kit). This effect is primarily explained by the reduced number of kits used per month in the silver group secondary to the lower frequency of dressing change at baseline and the reduced number of patients developing a DLI. Standard shipping of the control kit is 10 per month, and standard shipping of the silver kit is 5 per month. Additionally, patients who developed infections and drainage tended to change their dressing more often during the infection phase, thus driving their monthly driveline dressing cost.

## Study limitations

The study was limited by its single-center design and its small enrollment, which limits its predictive power. We did not investigate other variables that could influence DLI rates, such as nutrition status, gender, caregiver relationship, etc. There was a learning curve associated with the usage of the silver kit, leading us to modify the dressing protocol. The follow-up was limited to the 18-month point at which the study was halted secondary to the results analysis and the apparent superiority of the silver protocol. All patients were switched to the silver kit dressing thereafter.

## Conclusions

A weekly driveline dressing protocol that includes a non-activated silver patch provided protection against DLIs in our cohort. Demonstration of these findings in a larger, multicenter patient population to offer statistical significance would validate these results and help further advance the field.

## Conflicts of Interest statement

The authors declare the following financial interests/personal relationships, which may be considered as potential competing interests: Sylvie Baudart reports financial support was provided by the International Consortium of Circulatory Assist Clinicians. Sylvie Baudart reports a relationship with Medtronic that includes: speaking and lecture fees and travel reimbursement. Liviu Klein reports a relationship with Abbott that includes: speaking and lecture fees and travel reimbursement. If there are other authors, they declare that they have no known competing financial interests or personal relationships that could have appeared to influence the work reported in this paper.
